# 1-Methylhistidine attenuates early myocardial inflammation in type 3 cardiorenal syndrome through modulation of the p105/p50 NF-κB axis

**DOI:** 10.3389/fphar.2026.1974807

**Published:** 2026-10-14

**Authors:** Jiakun Zhang, Qian Wang, Xingyi Tu, Han Zhou, Shihui Wang, Jingjie Li

**Affiliations:** 1 Department of Cardiology, The First Affiliated Hospital of Harbin Medical University, Harbin Medical University, Harbin, Heilongjiang, China; 2 Department of Cardiology, The Fourth Affiliated Hospital of Harbin Medical University, Harbin Medical University, Harbin, Heilongjiang, China

**Keywords:** 1-methylhistidine, myocardial inflammation, NFKB1/p105, NF-κB signaling, type 3 cardiorenal syndrome

## Abstract

Type 3 cardiorenal syndrome (CRS3) is characterized by cardiac injury or dysfunction secondary to acute kidney injury (AKI), yet the contribution of AKI-associated metabolic remodeling to early myocardial inflammation remains poorly understood. Here, paired serum and myocardial untargeted metabolomics identified 1-methylhistidine (1-MH) as a metabolite elevated in both compartments, with the highest variable importance in projection score in serum. Exogenous 1-MH improved the E/A ratio and attenuated myocardial inflammation and terminal deoxynucleotidyl transferase dUTP nick-end labeling (TUNEL) positivity in rats with CRS3. Similar protective effects were observed in a separate post-ischemic cohort in which 1-MH was administered immediately after renal reperfusion. In CRS serum-exposed H9c2 cells, 1-MH reduced reactive oxygen species-associated fluorescence, malondialdehyde content, and intracellular Fe^2+^ levels. Transcriptomic profiling showed that 1-MH suppressed inflammatory signaling, accompanied by changes in oxidative metabolism. Integration of these transcriptomic signatures with target prediction prioritized NFKB1/p105, while ensemble docking identified recurrent candidate interaction regions within p105. Cellular thermal shift assays showed a concentration-related increase in p105 thermal stability following 1-MH treatment. 1-MH increased p105 abundance while reducing total p50 abundance and nuclear p50 accumulation. p105 overexpression increased p50 abundance and the expression of inflammatory transcripts and proteins suppressed by 1-MH. These findings demonstrate that 1-MH attenuates early myocardial inflammation in CRS3 and reduces oxidative stress-related changes in CRS serum-exposed H9c2 cells. These functional results support modulation of the p105/p50 NF-κB axis and identify p105 as a molecular link between AKI-associated metabolic remodeling and myocardial inflammatory signaling.

## 1 Introduction

Type 3 cardiorenal syndrome (CRS3), also termed acute renocardiac syndrome, refers to acute cardiac injury or dysfunction secondary to acute kidney injury (AKI) (Ronco et al., 2008; Ronco et al., 2010; Bagshaw et al., 2013; Okpara et al., 2024). Long-term survival among patients with CRS3 requiring intensive care has been reported to be below 50% (Drubel et al., 2022). AKI affects organs beyond the kidneys, including the heart, lung, liver, brain, and intestine. Circulating mediators and tissue stress responses emerge before overt remote-organ failure develops (Yap and Lee, 2012; Doi and Rabb, 2016; Lee et al., 2018; Faubel, 2023). Current explanations for CRS3 focus on volume and hemodynamic disturbances, neurohormonal activation, electrolyte and acid–base abnormalities, uremic toxins, and myocardial oxygen imbalance (Bagshaw et al., 2013; Zannad and Rossignol, 2018; Okpara et al., 2024). Yet the underlying pathophysiological mechanisms remain poorly defined.

AKI triggers systemic inflammation through molecular signals that link renal damage to inflammatory and metabolic stress in distant organs (Yap and Lee, 2012; Doi and Rabb, 2016; Lee et al., 2018; Faubel, 2023). Renal tissue damage activates pattern-recognition pathways, recruits immune cells, and promotes the release of cytokines and chemokines into the circulation (Doi and Rabb, 2016; Doi and Rabb, 2016). These systemic changes promote endothelial injury and sustained inflammatory responses in remote tissues. In CRS3, inflammation interacts with oxidative stress, ischemia–reperfusion injury, immune dysregulation, and neurohormonal activation (Morgan and Liu, 2011; Clementi et al., 2015). Inflammatory pathways, including TNF, IL-1, IL-17, and MAPK signaling, converge on NF-κB-dependent transcriptional regulation (Sanz et al., 2010; Hayden and Ghosh, 2012; Liu et al., 2017). NF-κB drives expression of inflammatory cytokines, chemokines, and inducible enzymes, among them TNF-α, IL-1β, IL-6, CXCL-family genes, and *Ptgs2*/COX-2 (Hayden and Ghosh, 2012; Liu et al., 2017; Fiordelisi et al., 2019). Pharmacological blockade of NF-κB nuclear transport suppresses stimulus-induced nuclear translocation and inflammatory gene expression (Lin et al., 1995). NF-κB thus bridges AKI-driven systemic inflammation and the myocardial inflammatory response.

NFKB1/p105 has dual functions in the NF-κB system: it is the precursor of p50 and an IκB-like regulatory protein (Liou et al., 1992; Rice et al., 1992; Savinova et al., 2009). Its N-terminal Rel homology region, retained in mature p50, mediates DNA binding and dimerization, whereas the C-terminal ankyrin-repeat region contributes to cytoplasmic retention of NF-κB complexes (Liou et al., 1992; Rice et al., 1992). Changes in p105 stability or processing can therefore alter p50 availability, the composition of nuclear NF-κB complexes, and downstream inflammatory transcription (Waterfield et al., 2003; Beinke et al., 2004; Perkins, 2007; Savinova et al., 2009; Yang et al., 2012). In the cardiovascular system, NF-κB signaling links inflammatory stimuli to cytokine production, cardiomyocyte stress, adverse remodeling, apoptotic signaling, and heart failure-related transcriptional programs (Gordon et al., 2011; Hamid et al., 2011; Maier et al., 2012; Liu et al., 2017; Fiordelisi et al., 2019). Because p105 regulates both p50 generation and cytoplasmic NF-κB retention, shifts in the p105/p50 balance influence how early systemic stress is translated into myocardial inflammation during CRS3.

Beyond inflammation, AKI disrupts mitochondrial and oxidative metabolism and reshapes the circulating biochemical milieu to which distant organs are exposed (Doi and Rabb, 2016; Tang et al., 2021). Oxidative stress and impaired cardiac energy metabolism have been detected as early as 4 h after ischemic AKI (Fox et al., 2019). By 72 h, renal ischemia–reperfusion has been linked to Drp1-dependent mitochondrial fission, cardiomyocyte apoptosis, and functional cardiac impairment (Sumida et al., 2015). Galectin-3 signaling (Prud’homme et al., 2019), histone deacetylase activity (Soranno et al., 2021), and Hippo pathway activation by circulating reactive oxygen species (Han et al., 2024) further link AKI-associated systemic stress to subsequent cardiac dysfunction. Metabolites can act as mediators of cellular responses rather than serving solely as markers of injury. Whether AKI-associated metabolic remodeling generates metabolites capable of modulating early myocardial inflammation in CRS3 remains unknown.

1-Methylhistidine (1-MH) is a histidine-related metabolite whose abundance is influenced by dietary intake and renal handling (Myint et al., 2000; Wishart et al., 2022). In methylglyoxal-exposed cells, 1-MH partially restored epithelial and endothelial barrier resistance (Wetzel et al., 2024), showing biological activity beyond its role as a metabolic marker. Reduced 1-MH abundance has also been reported among the metabolic alterations associated with serum exosome-induced endothelial dysfunction in diabetic microvascular disease (Yang et al., 2022). Whether 1-MH abundance changes after AKI, whether it influences early myocardial inflammation, and which molecular mechanisms underlie these effects remain unknown.

Paired serum and myocardial untargeted metabolomics in a rat model at 24 h identified 1-MH for functional evaluation. We assessed its effects *in vivo* and in CRS serum-exposed H9c2 cells, then integrated myocardial transcriptomics with *in silico* target prediction to investigate the molecular mechanisms involved. The prioritized target was then examined by ensemble docking and CETSA, followed by immunoblotting and p105 overexpression to assess the functional involvement of the p105/p50 NF-κB axis. We asked whether 1-MH protects against early myocardial injury in CRS3 and whether this signaling axis contributes to its effects.

## 2 Materials and methods

### 2.1 Animal model establishment

Male Wistar rats (6–8 weeks old, 160–230 g) were housed under specific pathogen-free conditions at 21 °C ± 1 °C and 50%–70% relative humidity under a 12-h light/12-h dark cycle, with free access to standard chow and water. Animals were randomly assigned to the experimental groups. An early CRS3 model was established by bilateral renal ischemia–reperfusion as previously described (Sumida et al., 2015; Fox et al., 2019). After induction with 3% isoflurane, anesthesia was maintained with 1.5% isoflurane. A midline abdominal incision was made to expose both kidneys, and the renal pedicles were clamped with sterile microaneurysm clamps for 60 min. Renal ischemia was confirmed by darkening of the kidney surface. After clamp removal, reperfusion was verified visually. Before wound closure, 0.25% bupivacaine (2 mg/kg) was infiltrated locally around the incision site, and the incision was then closed in layers. Postoperative analgesia was provided with subcutaneous butorphanol (1 mg/kg every 6 h). All animals undergoing surgery received the same analgesic regimen. Sham animals underwent the same surgical procedure without renal pedicle clamping.

1-MH (CAS 332-80-9) was dissolved in sterile normal saline and administered via the tail vein at 20 mg/kg 12 h before renal ischemia or the corresponding sham procedure. Control animals received an equal volume of saline. The prophylactic cohort included four groups: Sham + Saline, Sham + 1-MH, CRS + Saline, and CRS + 1-MH. A separate post-ischemic cohort included Sham, CRS, and CRS + 1-MH groups (n = 5 per group). In this cohort, 1-MH (20 mg/kg) was administered via the tail vein immediately after renal reperfusion. Sham and CRS rats received an equal volume of saline at the corresponding time point. The surgical procedure, 1-MH dose, administration route, and 24-h endpoint were otherwise identical to those in the prophylactic cohort. All animals in the post-ischemic cohort underwent echocardiography at 24 h before tissue collection. For brevity, rats subjected to bilateral renal ischemia–reperfusion are referred to as CRS rats throughout the manuscript. Twenty-four hours after reperfusion or the corresponding sham procedure, cardiac function was evaluated by echocardiography under isoflurane anesthesia (3% for induction and 1.5% for maintenance). While anesthesia was maintained, blood was collected from the abdominal aorta for serum preparation, followed by exsanguination and immediate left ventricular tissue harvest.

The study protocol was reviewed and approved by the Animal Care and Use Committee of the First Affiliated Hospital of Harbin Medical University (approval no. 2023172). All procedures were performed in accordance with institutional guidelines for the care and use of laboratory animals.

### 2.2 Echocardiography and serum biochemistry

Cardiac function was evaluated by transthoracic echocardiography using a Philips ultrasound system under isoflurane anesthesia (3% for induction and 1.5% for maintenance). Standardized views were acquired to assess left ventricular systolic and diastolic parameters. Image acquisition and analysis were performed with group allocation concealed from the investigator. Serum blood urea nitrogen (BUN) and creatinine were measured using an automated biochemical analyzer in the institutional clinical laboratory. For the time-course study, 0.25 mL of blood was collected serially from the same five rats at 6, 12, 24, and 48 h for BUN and creatinine measurements. Serum B-type natriuretic peptide (BNP) and cardiac troponin I (cTnI) were measured using rat-specific enzyme-linked immunosorbent assay (ELISA) kits (Jianglai Biotechnology, Shanghai, China). Values below the lower limit of detection were considered non-quantifiable.

### 2.3 Histology and TUNEL staining

Left ventricular tissue was fixed in 4% paraformaldehyde, embedded in paraffin, sectioned, and stained with hematoxylin and eosin (H&E). TUNEL-positive cells were detected in paraffin-embedded myocardial sections using a green-fluorescence TUNEL Cell Apoptosis Detection Kit (catalog no. SC223-02; Seven Biotech, Beijing, China). Nuclei were counterstained with 4′,6-diamidino-2-phenylindole (DAPI). Images were captured with a Zeiss microscope (Carl Zeiss, Oberkochen, Germany). Histological evaluation and TUNEL quantification were performed without knowledge of group allocation. Multiple randomly selected microscopic fields were analyzed for each animal, and the mean value for each animal was used for statistical analysis. Five animals per group were randomly selected from the seven animals used for the 24-h *in vivo* analyses for TUNEL staining and myocardial cytokine measurements. All five animals per group in the post-ischemic cohort were included in the TUNEL and myocardial cytokine analyses.

### 2.4 ELISA and cell-based oxidative stress assays

Myocardial IL-6 and TNF-α levels were measured in left ventricular tissue using rat-specific ELISA kits (IL-6, catalog no. RAB0311, Sigma-Aldrich; TNF-α, catalog no. KRC3011, Invitrogen). Tissue cytokine levels were normalized to total protein and expressed as pg/mg protein. Each biological sample was analyzed in technical triplicate, and the triplicate mean was treated as one biological replicate. Intracellular reactive oxygen species (ROS) in H9c2 cells were measured using a DCFH-DA-based Reactive Oxygen Species Assay Kit (catalog no. S0034S; Beyotime Biotechnology, Shanghai, China). Nuclei were counterstained with Hoechst, and fluorescence images were acquired under identical imaging settings. For each image, the median background intensity was subtracted from the green fluorescence channel, and the mean background-corrected 2′,7′-dichlorofluorescein (DCF) intensity was calculated. Five independent experiments were analyzed. Malondialdehyde (MDA) content was measured using a Malondialdehyde Content Assay Kit (catalog no. AKFA013C; Boxbio Science and Technology, Beijing, China) and expressed as nmol/10^6^ cells. Intracellular Fe^2+^ levels were determined using a Ferrous Ion Content Assay Kit (catalog no. AKIC004M; Boxbio Science and Technology, Beijing, China) and normalized to the mean value of the WT group. Both assays were performed with five biological replicates per group, and three technical wells were averaged for each biological replicate. The cell experiments included four groups: WT control, CRS, CRS +50 μM 1-MH, and CRS +100 μM 1-MH. WT control cells were cultured with 5% fetal bovine serum and 5% serum from Sham rats, whereas CRS cells were cultured with 5% fetal bovine serum and 5% serum from CRS rats.

### 2.5 Untargeted metabolomics

Untargeted metabolomic profiling was performed on paired serum and left ventricular tissue collected from the same randomly selected Sham and CRS rats (*n* = 4 biological replicates per group). Serum and myocardial samples were analyzed separately. Samples were extracted with methanol, vacuum-dried, reconstituted in 80% methanol/water containing 4 ppm 2-chloro-L-phenylalanine, and filtered through 0.22 μm PTFE membranes. Liquid chromatography–mass spectrometry (LC–MS) analysis was performed using a Vanquish ultra-high-performance liquid chromatography (UHPLC) system coupled to a Q Exactive mass spectrometer with electrospray ionization (Thermo Fisher Scientific, Waltham, MA, United States). Chromatographic separation used an ACQUITY UPLC HSS T3 column (2.1 × 100 mm, 1.8 μm; Waters, Milford, MA, United States) at 40 °C with a flow rate of 0.3 mL/min and an injection volume of 2 μL. Positive- and negative-ion modes were acquired separately over an m/z range of 100–1,000 using data-dependent MS/MS, with MS1 and MS/MS resolutions of 70,000 and 17,500, respectively, and a normalized collision energy of 30 eV. Raw data were converted to mzXML format using ProteoWizard MSConvert (version 3.0.8789) and processed separately for each ionization mode in XCMS (version 3.12.0) for feature detection, retention-time correction, alignment, filtering, and total peak-area normalization. Metabolites were annotated against HMDB, MassBank, LIPID MAPS, mzCloud, KEGG, and an in-house spectral library using accurate mass and available MS/MS fragmentation data. Principal component analysis (PCA), partial least squares discriminant analysis (PLS-DA), and orthogonal partial least squares discriminant analysis (OPLS-DA) were performed using ropls (version 1.22.0). Differential metabolites were prioritized using a variable importance in projection (VIP) score >1.0 together with *P* < 0.05. Cross-compartment prioritization considered VIP ranking, fold-change magnitude, and concordance in the direction of change between serum and myocardium. Annotation and reporting followed established metabolomics guidelines (Sumner et al., 2007; Wishart et al., 2022).

### 2.6 RNA sequencing and pathway analysis

Total RNA was extracted from left ventricular tissue of CRS and CRS + 1-MH rats (n = 4 biological replicates per group). RNA integrity and genomic DNA contamination were evaluated by agarose gel electrophoresis, and RNA concentration and purity were measured using a NanoDrop spectrophotometer. Poly(A)-selected mRNA was enriched with oligo (dT) beads for library preparation. Libraries passing quality control were sequenced on an Illumina NovaSeq 6000 platform (Illumina, San Diego, CA, United States) with paired-end 150-bp reads. Adapter sequences and low-quality reads were removed before alignment to the *Rattus norvegicus* mRatBN7.2 reference genome (Ensembl release 108) using HISAT2 (v2.2.1). Gene counts were generated with HTSeq (union mode) and analyzed using DESeq2 in R. Genes with an absolute log_2_ fold change >1 and a Benjamini–Hochberg-adjusted P value < 0.05 were considered differentially expressed. Gene Ontology (GO) and Kyoto Encyclopedia of Genes and Genomes (KEGG) enrichment analyses were performed using clusterProfiler. Gene set enrichment analysis (GSEA) was conducted using fgsea with 1,000 permutations. Selected transcriptomic findings were further examined at the mRNA and protein levels using RT-qPCR and immunoblotting as orthogonal experimental follow-up.

### 2.7 Western blotting and RT-qPCR

Total protein was extracted from H9c2 cells using ice-cold lysis buffer supplemented with protease and phosphatase inhibitors. Protein concentration was determined by a bicinchoninic acid (BCA) assay. Equal amounts of protein were separated by sodium dodecyl sulfate-polyacrylamide gel electrophoresis (SDS-PAGE) and transferred onto polyvinylidene fluoride (PVDF) membranes. Membranes were blocked with 5% nonfat milk or bovine serum albumin (BSA) before overnight incubation at 4 °C with primary antibodies against NF-κB1 p105/p50 (catalog no. 13586; Cell Signaling Technology), COX-2 (catalog no. YM8171; Immunoway), IL-1β (catalog no. GB11113; Servicebio), ACSL4 (catalog no. GB115608; Servicebio), and GAPDH (catalog no. GB15004; Servicebio). After incubation with horseradish peroxidase-conjugated secondary antibodies, protein bands were visualized by enhanced chemiluminescence. The 31-kDa IL-1β band was quantified as IL-1β. Band intensities were quantified in ImageJ and normalized to GAPDH. For p105/p50 immunoblotting performed on separate membranes, a shared bridge sample was included on each membrane for signal normalization before between-membrane comparisons (Degasperi et al., 2014). Total RNA was extracted from H9c2 cells using the SevenFast Total RNA Extraction Kit for Cells (catalog no. SM132; Seven Biotech, Beijing, China), followed by reverse transcription into cDNA. The expression of *Ptgs2*, *Cxcl2*, and *Il1b* was measured by reverse transcription quantitative PCR (RT-qPCR) using gene-specific primers. Relative mRNA expression was calculated using the 2^−ΔΔCT^ method with *Gapdh* as the reference gene. Primer sequences are listed in Supplementary Table S1.

### 2.8 Immunofluorescence and p50 nuclear translocation

H9c2 cells cultured on coverslips were fixed with 4% paraformaldehyde, permeabilized with Triton X-100, and blocked with bovine serum albumin-containing buffer. Cells were incubated overnight at 4 °C with an anti-p50 primary antibody (catalog no. 13586; Cell Signaling Technology, Danvers, MA, United States), followed by incubation with a fluorophore-conjugated secondary antibody and counterstaining with DAPI. Images were acquired under identical imaging conditions for all groups. Nuclear regions were defined by DAPI staining, and cytoplasmic regions were generated by subtracting the nuclear mask from the corresponding whole-cell area. Nuclear and cytoplasmic p50 fluorescence intensities were quantified to calculate the nuclear-to-cytoplasmic fluorescence ratio. Multiple randomly selected fields were analyzed in each independent experiment without knowledge of group allocation, and each independent experiment was treated as one biological replicate.

### 2.9 Target prediction, ensemble-based docking, and analysis of candidate interaction regions

Candidate protein targets of 1-MH were predicted using SuperPred 3.0 and ranked by predicted probability; the corresponding model accuracy was also recorded for each target (Gallo et al., 2022). NFKB1/p105 was prioritized for structural analysis because it ranked first among the predicted targets and was consistent with the transcriptomic findings. An ensemble of 60 independently generated AlphaFold 3 NFKB1/p105 structural models was assembled with AlphaFold 3 (Abramson et al., 2024) using reviewed UniProt reference sequences (The UniProt Consortium, 2025) for *R. norvegicus* (Q63369), *Mus musculus* (P25799), and *Homo sapiens* (P19838). The ensemble included 30 rat, 15 mouse, and 15 human models. The three-dimensional structure of 1-MH was retrieved from PubChem (Kim et al., 2025) and prepared in PDBQT format. For all docking analyses, receptor structures were standardized to a single full-length p105 chain.

Blind docking was performed across the 30 rat p105 models using a search box covering the full protein with an 8 Å margin. Exhaustiveness was set to 32 and num_modes to 20. For each model, the center-of-mass coordinates of the top-ranked ligand pose were recorded. To compare docking locations across models, the ANK region (rat residues 545–743, Cα atoms) was aligned to a common rat reference using Kabsch superposition, and the ligand coordinates were transformed accordingly. These coordinates were then clustered using density-based spatial clustering of applications with noise (DBSCAN), with min_samples = 3 and ε values of 6, 8, and 10 Å. Two clusters were consistently recovered across all three ε settings and were designated C1 and C2 for subsequent focused docking. Before focused docking, all 60 receptor models were superposed onto the same rat reference structure using the ANK region, allowing the C1 and C2 search-box centers to be applied consistently across the ensemble. Focused docking was performed using AutoDock Vina v1.2.7 (Eberhardt et al., 2021), with exhaustiveness set to 32, num_modes to 20, energy_range to 3 kcal/mol, and identical 22 × 22 × 22 Å search boxes centered on either C1 or C2. Each receptor–region pair underwent 10 independent docking runs using different Vina random seeds. The top-ranked score from each run was retained, and the median of the 10 scores served as the representative score for that receptor–region pair. The repeated runs were treated as technical replicates and were not considered independent observations for statistical inference. Paired differences were defined as Δscore = C1 − C2, with negative values indicating a more favorable score for C1 than for C2. Overall and species-specific differences were evaluated using paired Wilcoxon signed-rank tests. Hodges–Lehmann shift estimates were reported with 95% CIs obtained by paired bootstrap resampling (10,000 iterations). Species-specific P values were adjusted using the Holm method.

Technical stability was assessed for each receptor–region pair using the range and interquartile range (IQR) of the 10 top-ranked docking scores. For contact analysis, the top-ranked pose from a fixed-seed (seed = 0) focused docking run was used for each receptor–region pair. Ligand–protein contacts were defined by a minimum heavy-atom distance of ≤4.0 Å. Following sequence alignment, homologous mouse and human residues were mapped to a unified rat reference numbering scheme, and contact frequencies were calculated across the full ensemble and within each species. Structural confidence near C1 was assessed using AlphaFold 3 confidence metrics. For each NFKB1/p105 model, the mean pairwise PAE between the RHD-CT (residues 241–400) and ANK (residues 545–743) regions was calculated in both region orientations and averaged to assess uncertainty in their relative interdomain positioning. Local structural confidence was evaluated using the per-residue pLDDT profile of the same representative rat NFKB1/p105 model shown in the PAE map. The RHD-CT and ANK regions and selected C1-associated residues were marked on this profile. Structural confidence at C2 was further assessed descriptively using local pLDDT values for recurrent contact residues and bidirectional mean PAE for the ANK–Death, ANK–C-terminal, and Death–C-terminal region pairs. Complete SuperPred results are provided in Supplementary Table S3. Docking stability measures, residue-contact analyses, and RHD-CT–ANK interdomain PAE results are provided in Supplementary Figure S2 and Supplementary Table S4.

### 2.10 Cellular thermal shift assay (CETSA)

CETSA was performed to evaluate the effect of 1-MH on the thermal stability of intracellular p105, as previously described by Martinez Molina et al. (2013). H9c2 cells were cultured for 24 h in medium containing 5% fetal bovine serum and 5% serum from CRS rats in the presence of vehicle, 50 μM 1-MH, or 100 μM 1-MH. Cells were then harvested, divided into equal aliquots, and either maintained at room temperature (RT) or heated at 40, 45, 50, 55, or 60 °C for 3 min. Following freeze–thaw lysis, soluble protein fractions were collected by centrifugation and analyzed by western blotting using an NF-κB1 p105 antibody (catalog no. 4717; Cell Signaling Technology). Within each treatment group, p105 band intensity at each temperature was normalized to the corresponding RT control. Three independent experiments were performed.

### 2.11 Cell culture and p105 overexpression

H9c2 rat cardiomyoblasts (ABCell, catalog no. AC601) were cultured in Dulbecco’s modified Eagle’s medium (DMEM) supplemented with 10% fetal bovine serum at 37 °C in a humidified atmosphere containing 5% CO_2_ and 95% air. Cells in the logarithmic growth phase were used for subsequent experiments. To establish the *in vitro* CRS-associated injury model, the culture medium was replaced with DMEM containing 5% fetal bovine serum and 5% serum from CRS rats for 24 h. Cells cultured in DMEM supplemented with 5% fetal bovine serum and 5% serum from Sham rats served as the WT group. For analysis of p105/p50 expression, cells were assigned to WT, WT + 1-MH, CRS, and CRS + 1-MH groups. Where indicated, 1-MH (50 μM) was added to the culture medium during serum treatment. For p105 overexpression, cells were seeded in six-well plates and transiently transfected with 1.5 μg/well of a rat *Nfkb1* overexpression plasmid or the corresponding empty vector using Lipofectamine 3,000 (Thermo Fisher Scientific, Waltham, MA, United States). Forty-eight hours after transfection, the culture medium was replaced with Sham or CRS serum-containing medium in the presence or absence of 50 μM 1-MH, followed by an additional 24 h of incubation before sample collection for immunoblotting, RT-qPCR, or immunofluorescence analysis. All non-overexpression conditions received the corresponding empty vector; the vector designation was omitted from abbreviated figure labels for clarity. Detailed plasmid information is provided in Supplementary Table S2.

### 2.12 Statistical analysis

Data are presented as mean ± standard deviation (SD) unless otherwise indicated. Statistical analyses were performed using R (version 4.3). For animal experiments, each animal was considered one biological replicate. The post-ischemic and prophylactic cohorts were analyzed separately, with no statistical comparisons between cohorts. For cell-based experiments, each independent experiment was treated as one biological replicate. Technical replicates and multiple microscopic fields were averaged before statistical analysis. Untargeted metabolomics and RNA sequencing each included four biological replicates per group. Two-group comparisons were performed using two-sided unpaired Student’s t tests when assumptions of normality and homogeneity of variance were satisfied. Comparisons among multiple groups were analyzed by one-way analysis of variance (ANOVA) followed by Tukey’s honestly significant difference (HSD) test. Welch’s ANOVA with Games–Howell *post hoc* testing or the Kruskal–Wallis test followed by Dunn’s multiple-comparison test was used when standard ANOVA assumptions were not met. Relative 1-MH abundance was compared between the Sham and CRS groups separately in serum and myocardium using the Mann–Whitney U test. DCF fluorescence intensity was analyzed by one-way ANOVA followed by Bonferroni-adjusted pairwise comparisons. RT-qPCR data were log_2_-transformed before statistical analysis. RNA sequencing data were analyzed using DESeq2 with Benjamini–Hochberg correction for differential expression. GO and KEGG enrichment analyses were performed using clusterProfiler, and GSEA was conducted using fgsea. Metabolomic multivariate analyses were conducted using ropls. Unless otherwise specified, all statistical tests were two-sided, and *P* < 0.05 was considered statistically significant.

## 3 Results

### 3.1 1-MH was prioritized as a cross-compartment metabolite in early CRS3

The overall experimental workflow is summarized in Figure 1A. Paired serum and myocardial metabolomic profiling at 24 h after reperfusion captured systemic and local metabolic responses within the same animals. Serum PCA partially separated Sham and CRS samples (Figure 1C). Of 361 annotated serum metabolites, 53 met the differential criteria (Figure 1D). Among the leading VIP-ranked metabolites, 1-methylhistidine (1-MH) had the highest serum VIP score (2.11) and was 3.03-fold higher in CRS than in Sham serum (Figures 1B,D; Supplementary Figure S1A). Myocardial PCA also partially separated the groups (Figure 1F). Of 280 annotated myocardial metabolites, 25 were differentially abundant (22 increased and 3 decreased; Figures 1E–G). 1-MH abundance was also higher in CRS myocardium (Supplementary Figure S1B). Cross-compartment prioritization integrated VIP rank, fold-change magnitude, and direction of change (Figure 1H). 1-MH met the screening criteria in both serum and myocardium and showed concordant changes across the two compartments. Based on its cross-compartment profile and high serum VIP rank, we prioritized 1-MH for subsequent functional evaluation.

**FIGURE 1:**
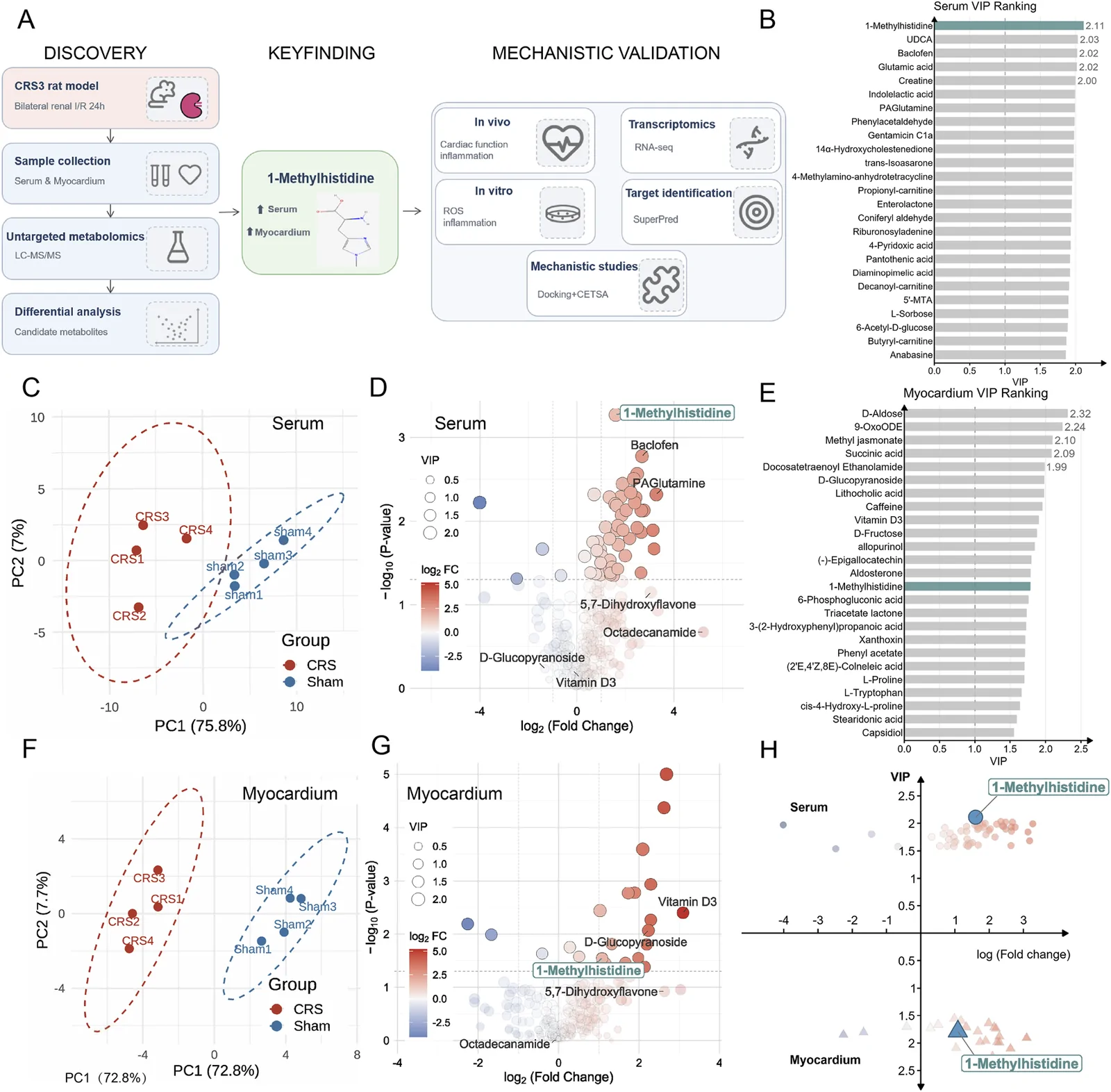
Cross-compartment prioritization of 1-MH in early CRS3. **(A)** Schematic overview of the workflow for metabolite identification, cross-compartment prioritization, and subsequent mechanistic investigation. **(B)** Top 25 VIP-ranked serum metabolites. **(C)** PCA score plot of serum metabolomic profiles from Sham and CRS rats. **(D)** Serum volcano plot highlighting 1-MH. **(E)** Top 25 VIP-ranked myocardial metabolites. **(F)** PCA score plot of myocardial metabolomic profiles from Sham and CRS rats. **(G)** Myocardial volcano plot highlighting 1-MH. **(H)** Cross-compartment prioritization of differential metabolites based on VIP ranking, fold-change magnitude, and concordance in the direction of change between serum and myocardium. Differential metabolites were screened using VIP >1.0 and P < 0.05. Metabolomic analyses were performed with n = 4 biological replicates per group. Relative 1-MH abundance in serum and myocardium is presented in Supplementary Figure S1. 1-MH, 1-methylhistidine; PCA, principal component analysis; VIP, variable importance in projection.

### 3.2 1-MH attenuated early myocardial inflammation in CRS3 and oxidative stress in CRS serum-exposed H9c2 cells

The animal experimental design and treatment timeline are shown in Figure 2A. In a time course, serum BUN and creatinine peaked 24 h after reperfusion (Figures 2B,C); all subsequent *in vivo* analyses were therefore conducted at this time point. At 24 h, BUN rose from 43.20 ± 3.86 mg/dL in Sham rats to 197.20 ± 8.94 mg/dL in CRS rats, and creatinine rose from 55.21 ± 9.04 to 206.25 ± 19.67 μmol/L (Figures 2E,F), confirming successful AKI induction. Serum BNP increased from 65.3 ± 4.6 pg/mL in Sham rats to 110.2 ± 18.3 pg/mL in CRS rats and fell to 77.5 ± 5.7 pg/mL after 1-MH treatment, whereas most cTnI values remained below the lower limit of detection. The E/A ratio declined from 1.326 ± 0.089 in Sham rats to 1.121 ± 0.036 in CRS rats and increased to 1.241 ± 0.042 following 1-MH treatment, whereas left ventricular ejection fraction (LVEF) and fractional shortening remained unchanged (Figures 2D,G–J). These findings demonstrated diastolic dysfunction with preserved systolic function. Gross cardiac morphology showed no obvious differences among the groups (Figure 2K). H&E staining revealed limited inflammatory cell infiltration without overt disruption of myocardial architecture (Figure 2L). 1-MH reduced serum BNP and improved diastolic function without significantly altering BUN or serum creatinine.

**FIGURE 2:**
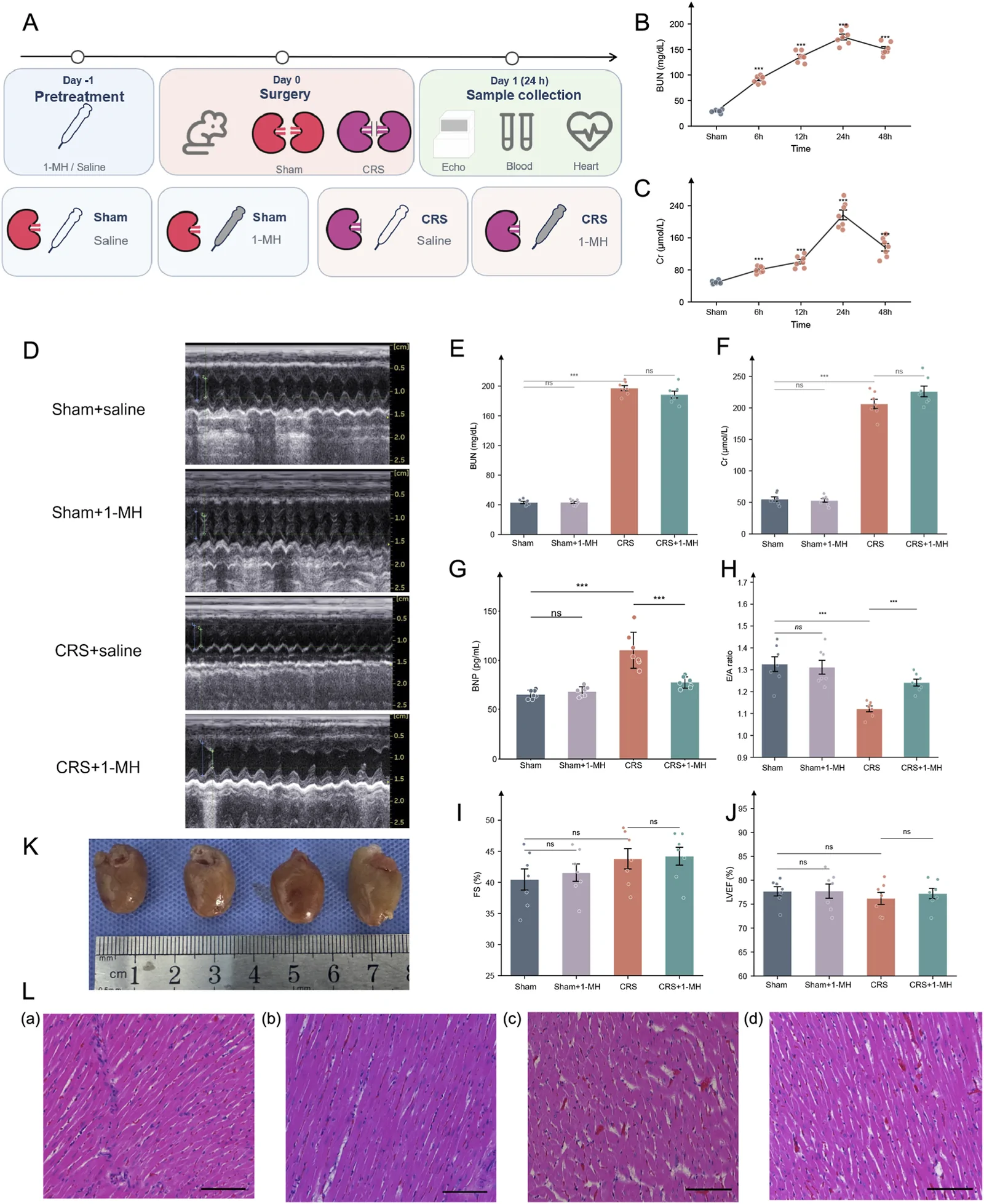
Establishment of AKI-induced early myocardial involvement in CRS3 at 24 h. **(A)** Schematic illustration of the animal experimental design and treatment timeline. **(B,C)** Time-course changes in serum blood urea nitrogen (BUN) and creatinine at 6, 12, 24, and 48 h after bilateral renal ischemia-reperfusion, obtained by serial sampling from the same five rats (0.25 mL per time point). **(D)** Representative echocardiographic images. **(E,F)** Serum BUN and creatinine concentrations measured 24 h after reperfusion in the Sham + Saline, Sham + 1-MH, CRS + Saline, and CRS + 1-MH groups. **(G)** Serum B-type natriuretic peptide (BNP) concentrations measured by ELISA. **(H)** Quantification of the E/A ratio. **(I,J)** Quantification of fractional shortening (FS) and left ventricular ejection fraction (LVEF). **(K)** Representative gross cardiac morphology. **(L)** Representative H&E-stained myocardial sections. Serum cardiac troponin I (cTnI) was measured by ELISA but is not shown because only a small number of samples exceeded the lower limit of detection. **(B,C)** show descriptive time-course data from n = 5 animals; **(E–J)** were obtained from n = 7 biological replicates per group and analyzed using one-way ANOVA followed by Tukey’s HSD test. Data are presented as mean ± SD. *P < 0.05, **P < 0.01; ns, not significant.

At the tissue level, 1-MH had no significant effect on myocardial TUNEL positivity, IL-6, or TNF-α levels in sham-operated rats. Myocardial TUNEL positivity rose from 2.00% ± 0.23% in Sham rats to 16.61% ± 2.08% in CRS rats and fell to 9.68% ± 0.53% after 1-MH treatment. Myocardial IL-6 and TNF-α levels rose in CRS rats and declined after 1-MH treatment (Figures 3A–D). We also assessed whether 1-MH exerted protective effects when administered immediately after renal reperfusion in a separate cohort. At 24 h, 1-MH increased the E/A ratio in CRS rats from 1.144 to 1.268 (Sham, 1.374), whereas fractional shortening and LVEF remained unchanged. Myocardial TUNEL positivity decreased from 15.26% to 10.84% after 1-MH treatment (Sham, 1.69%), and myocardial IL-6 and TNF-α levels were also lower (Supplementary Figure S3). In H9c2 cells, exposure to CRS serum increased DCF fluorescence intensity, MDA content, and intracellular Fe^2+^ levels. Both 50 and 100 μM 1-MH significantly reduced all three readouts relative to CRS serum alone, with greater reductions at 100 μM than at 50 μM (Figures 4A–D).

**FIGURE 3:**
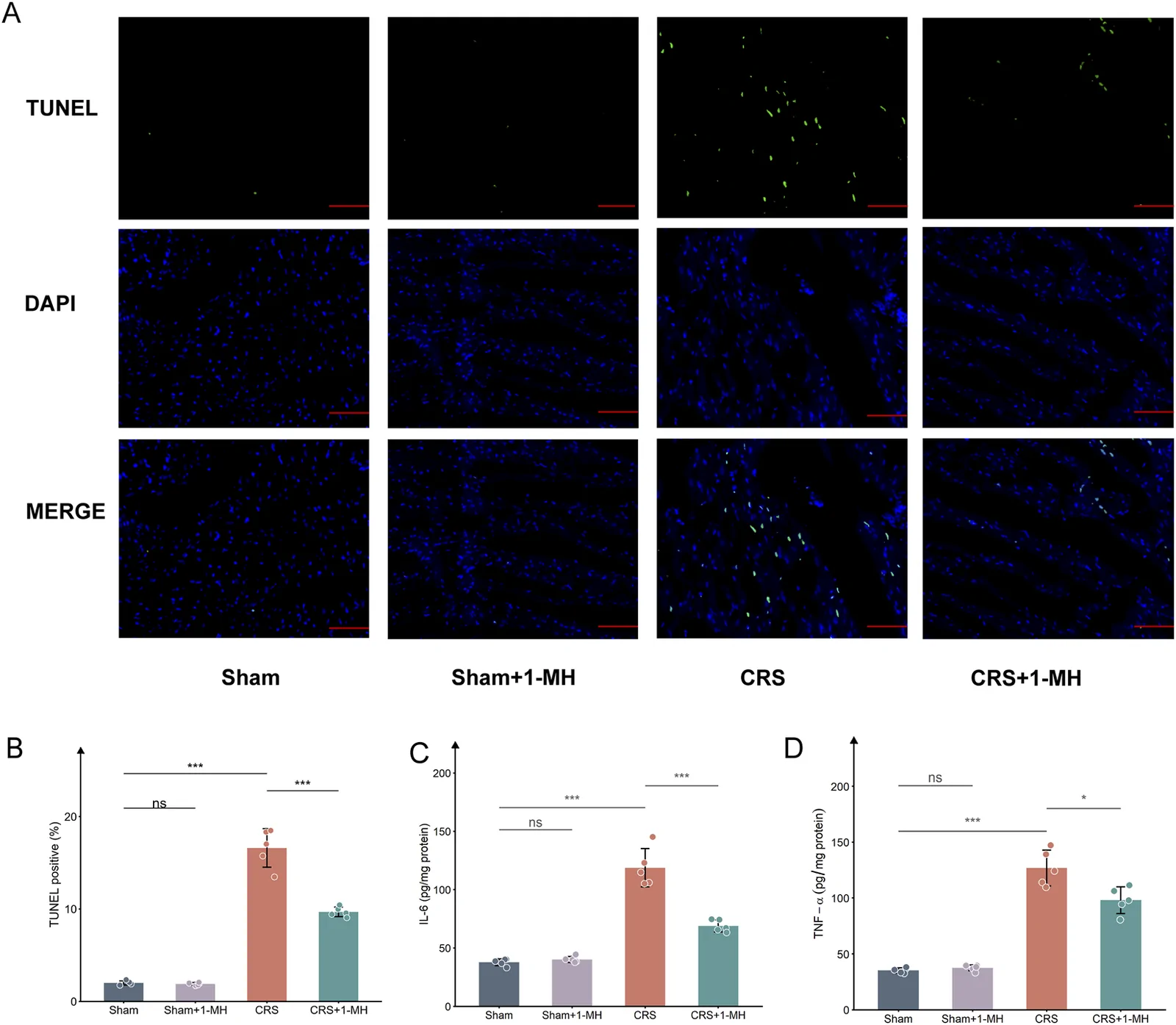
1-MH reduces myocardial TUNEL positivity and inflammatory cytokine levels in early CRS3. **(A)** Representative TUNEL fluorescence images of myocardial sections with DAPI counterstaining and merged images. Scale bars, 50 μm. **(B)** Quantification of TUNEL-positive myocardial cells. **(C,D)** Myocardial IL-6 and TNF-α levels measured by ELISA and normalized to total protein. **(B–D)** represent data from five animals per group. ELISA technical triplicates were averaged to yield one biological data point per animal. Data are shown as mean ± SD and were analyzed by one-way ANOVA with Tukey’s HSD test; *P < 0.05, **P < 0.01, ***P < 0.001; ns, not significant.

**FIGURE 4:**
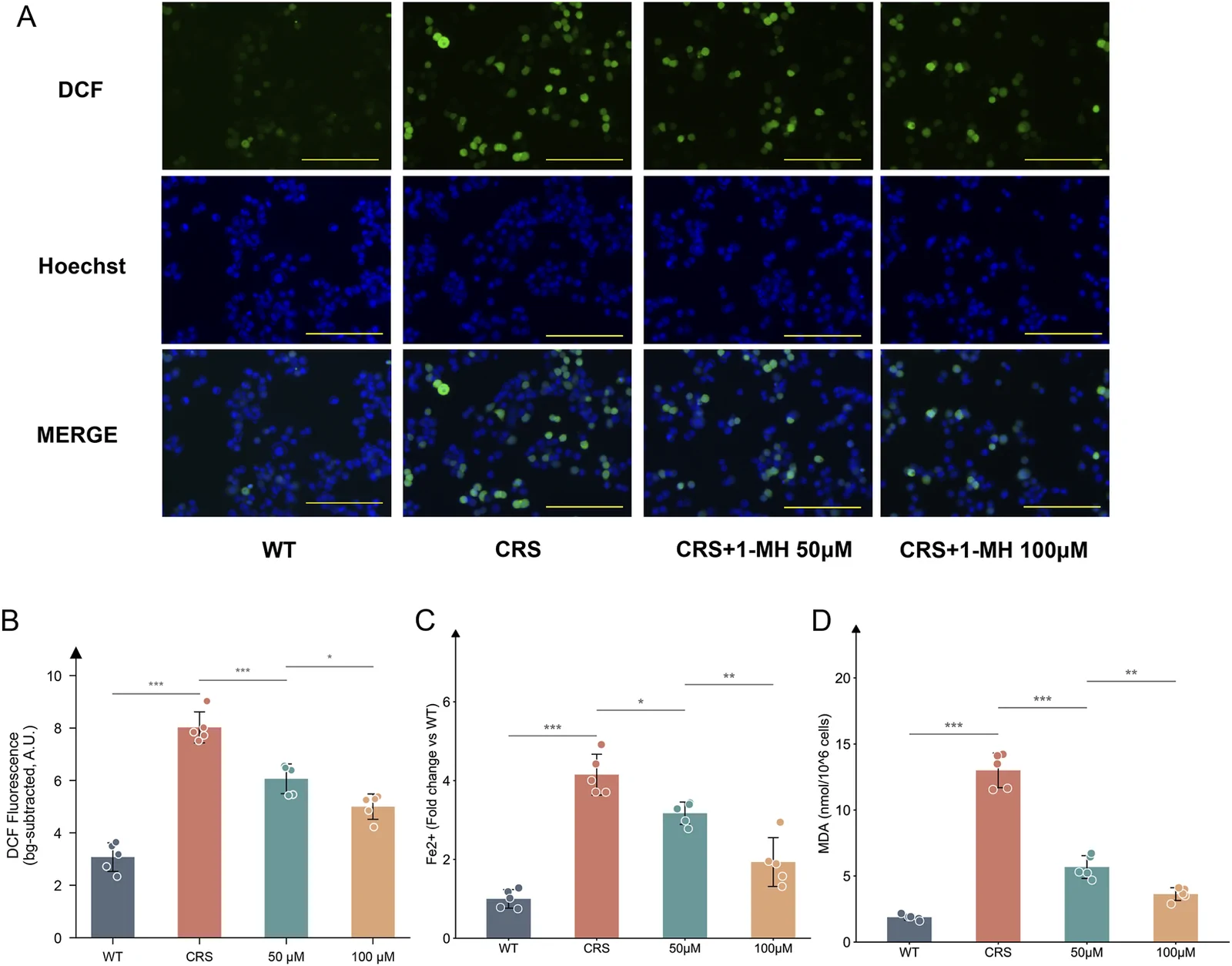
1-MH attenuates CRS serum-induced oxidative stress-related changes in H9c2 cells. Cells were examined after 24 h under WT, CRS, CRS + 1-MH (50 μM), and CRS + 1-MH (100 μM) conditions. **(A)** Representative fluorescence images showing DCF (green), Hoechst (blue), and merged channels. Scale bars, 50 μm. **(B)** Quantification of intracellular ROS fluorescence intensity. **(C)** Intracellular Fe^2+^ levels. **(D)** Malondialdehyde (MDA) concentrations. **(B)** represents five independent experiments per group. **(C,D)** represent five biological replicates per group, with three technical wells averaged to yield one biological data point. **(B)** was analyzed by one-way ANOVA with Bonferroni-adjusted pairwise comparisons; **(C,D)** were analyzed by one-way ANOVA with Tukey’s HSD test. Data are shown as mean ± SD; *P < 0.05, **P < 0.01, ***P < 0.001; ns, not significant.

### 3.3 1-MH suppressed inflammatory transcriptional programs and altered oxidative metabolism in CRS myocardium

RNA-seq profiling characterized the myocardial transcriptional response to 1-MH under CRS conditions. Of 12,835 detected genes, 272 were differentially expressed between the CRS + 1-MH and CRS groups (adjusted P < 0.05, |log_2_ fold change| > 1), comprising 98 genes upregulated and 174 genes downregulated in the CRS + 1-MH group relative to the CRS group (Figure 5A). KEGG enrichment analysis identified multiple inflammation-related pathways, including TNF, IL-17, NF-κB, MAPK, Toll-like receptor, JAK–STAT, NOD-like receptor, and chemokine signaling (Figure 5B); 19 of the top 20 enriched pathways were associated with inflammation or oxidative metabolism. GSEA corroborated these findings: inflammatory gene sets, including TNF, NF-κB, MAPK, JAK–STAT, NOD-like receptor, chemokine signaling, and cytokine–cytokine receptor interactions, were negatively enriched in the CRS + 1-MH group (Figure 5C). GO enrichment analysis revealed responses to lipopolysaccharide and oxygen-containing compounds, regulation of inflammation and NIK/NF-κB signaling, complement activation, nutrient responses, and apoptotic signaling (Figure 5D). The myocardial transcriptional response to 1-MH was characterized by suppression of inflammatory signaling, accompanied by broader changes in oxidative metabolism.

**FIGURE 5:**
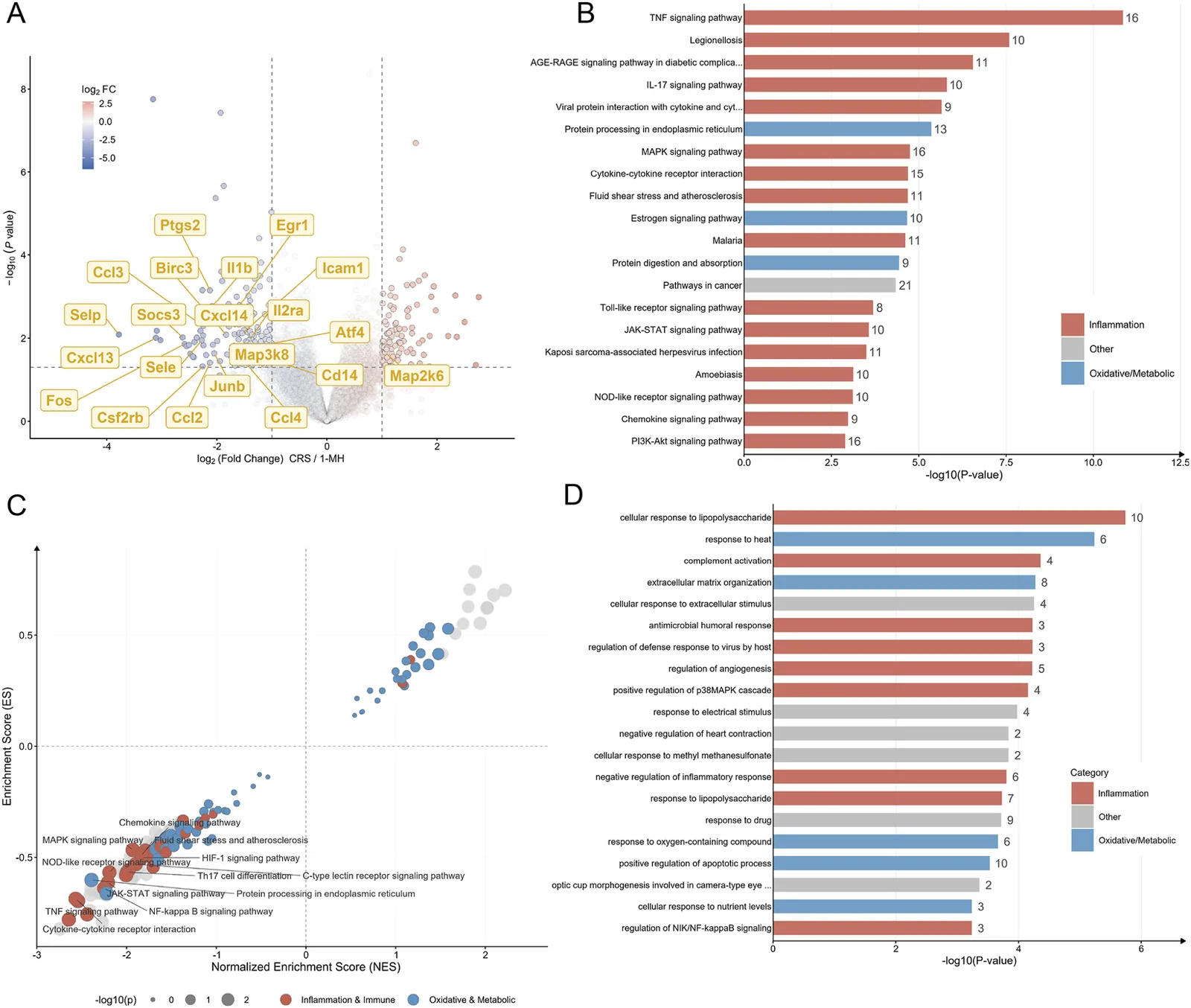
Transcriptomic analysis identifies inflammation-related responses and altered oxidative metabolism after 1-MH treatment in CRS myocardium. **(A)** Volcano plot of genes differentially expressed between the CRS + 1-MH and CRS groups, showing 272 differentially expressed genes (DEGs; 98 upregulated and 174 downregulated; |log_2_ fold change| > 1 and adjusted P < 0.05). **(B)** KEGG pathway enrichment analysis of the DEGs. **(C)** Gene set enrichment analysis (GSEA) showing changes in NF-κB, TNF, IL-17, and oxidative phosphorylation-related pathways. **(D)** Gene Ontology (GO) biological process enrichment analysis of the DEGs. RNA-seq analyses were performed with n = 4 biological replicates per group.

### 3.4 Computational prioritization of NFKB1/p105

SuperPred predictions were integrated with RNA-seq findings to prioritize candidate protein targets of 1-MH. NFKB1/p105 ranked first among the predicted targets (probability, 0.9909; SuperPred model accuracy, 0.9609; Figure 6A). Blind docking of 1-MH against 30 independently generated rat p105 models identified two recurrent candidate interaction regions, designated C1 and C2 (Figure 6B). We therefore compared these regions across a cross-species ensemble of 60 rat, mouse, and human p105 models. Across the full ensemble, C1 showed more favorable docking scores than C2 in 42 of 60 models. The median docking score for C1 was −4.77 kcal/mol (IQR: −4.97 to −4.52), versus −4.41 kcal/mol (IQR: −4.73 to −4.19) for C2. The paired Hodges–Lehmann shift estimate was −0.28 kcal/mol (95% CI: −0.41 to −0.16; P = 9.70 × 10^−5^; Figures 6C,D). This preference was strongest in the rat ensemble, where C1 showed more favorable docking scores in 24 of 30 models (Hodges–Lehmann estimate: −0.35 kcal/mol; 95% CI: −0.50 to −0.17; Holm-adjusted P = 0.0031); the corresponding differences did not reach significance in the mouse or human ensembles. We prioritized C1 for detailed structural interpretation and retained C2 as a secondary candidate interaction region. Recurrent C2 contacts involved the ANK, Death, and C-terminal portions of p105, with the C-terminal component showing lower local structural confidence and greater uncertainty in its relative positioning (Supplementary Table S4). In a representative rat NFKB1/p105 C1 docking pose, 1-MH was positioned near Asp303, Ser682, Arg684, and Thr718 within the predicted RHD-CT–ANK interdomain region (Figure 6E). Across the C1 ensemble, contact frequencies for these residues were 88.3%, 95.0%, 95.0%, and 93.3%, respectively (Supplementary Figure S2B-C; Supplementary Table S4). In the representative rat model, local pLDDT values were relatively high across the RHD-CT and ANK regions, including at the selected C1-associated residues. Across the ensemble, interdomain PAE nevertheless indicated remaining uncertainty in the relative orientation of the two regions (Supplementary Figure S2D-F).

**FIGURE 6:**
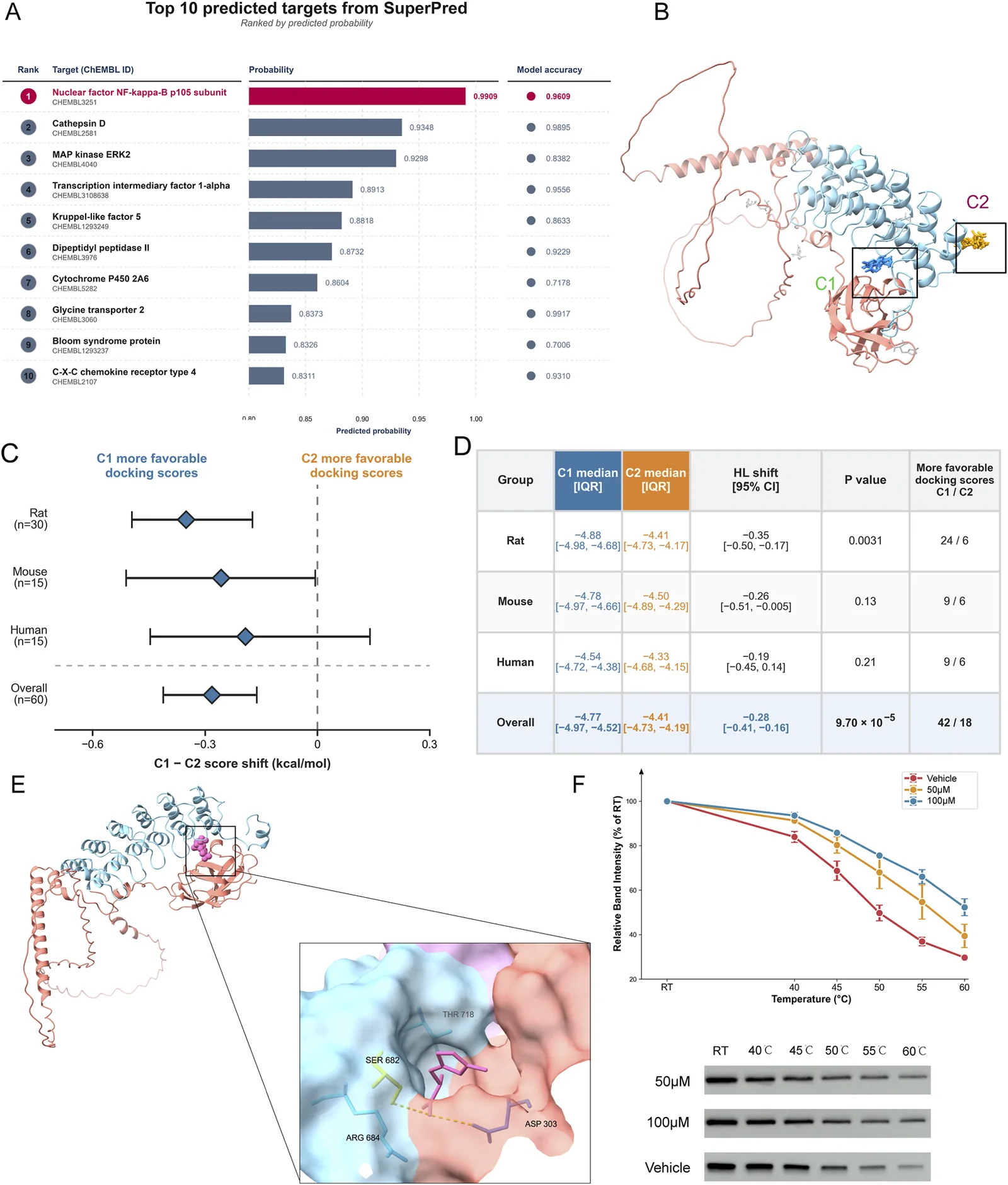
Integrated target prioritization and CETSA findings are consistent with intracellular p105 target engagement by 1-MH. **(A)** Top 10 protein targets predicted by SuperPred, ranked according to predicted probability. Model accuracy is shown for each target, with NFKB1/p105 ranked first. **(B)** Locations of the two recurrent candidate interaction regions, C1 and C2, on the AlphaFold 3-predicted rat NFKB1/p105 structure. **(C)** Cross-species comparison of paired docking score differences (C1 − C2) in rat, mouse, and human NFKB1/p105 models. Negative values indicate more favorable docking scores for C1 than for C2. Diamonds indicate Hodges–Lehmann shift estimates, and horizontal lines indicate the corresponding bootstrap 95% confidence intervals. **(D)** Species-stratified and overall focused-docking results. Across the complete ensemble, C1 showed more favorable docking scores than C2 in 42 of 60 models. Species-specific P values were adjusted using the Holm method. **(E)** Representative candidate interaction mode of 1-MH within the C1 region of rat p105. The enlarged view shows 1-MH positioned near Asp303, Ser682, Arg684, and Thr718 within the predicted RHD-CT–ANK interdomain region. **(F)** CETSA analysis of p105 in CRS serum-exposed H9c2 cells after 24 h treatment with vehicle, 50 μM 1-MH, or 100 μM 1-MH. Soluble p105 retained at room temperature (RT) and after heating at 40, 45, 50, 55, and 60 °C was normalized to the corresponding RT value. Data are presented as mean ± SEM from three independent experiments. CETSA showed a concentration-related increase in p105 thermal stability after 1-MH treatment. ANK, ankyrin-repeat region; CETSA, cellular thermal shift assay; RHD-CT, C-terminal portion of the Rel homology domain; SEM, standard error of the mean.

### 3.5 1-MH increased p105 thermal stability and modulated the p105/p50 NF-κB axis

We used CETSA to assess the thermal stability of intracellular p105 following 1-MH treatment. Treatment with 50 or 100 μM 1-MH increased soluble p105 retention after heating, with greater stabilization at 100 μM (Figure 6F). The concentration-related increase in p105 thermal stability supported intracellular p105 target engagement by 1-MH. 1-MH alone did not significantly alter basal p105 or p50 abundance in WT control cells. CRS serum increased both p105 and p50 abundance in H9c2 cells. In CRS-stimulated cells, co-treatment with 50 μM 1-MH further increased p105 abundance (WT control: 1.000 ± 0.218; CRS serum: 1.669 ± 0.218; CRS + 1-MH: 2.664 ± 0.429; Figures 7A–C), whereas p50 abundance decreased (WT control: 1.000 ± 0.157; CRS serum: 2.511 ± 0.461; CRS + 1-MH: 1.689 ± 0.329; Figures 7A–C). The reduction in total p50 was accompanied by a lower nuclear-to-cytoplasmic p50 ratio (Figures 7D,E). 1-MH increased p105 thermal stability, shifted the p105/p50 balance toward p105, and reduced nuclear p50 accumulation.

**FIGURE 7:**
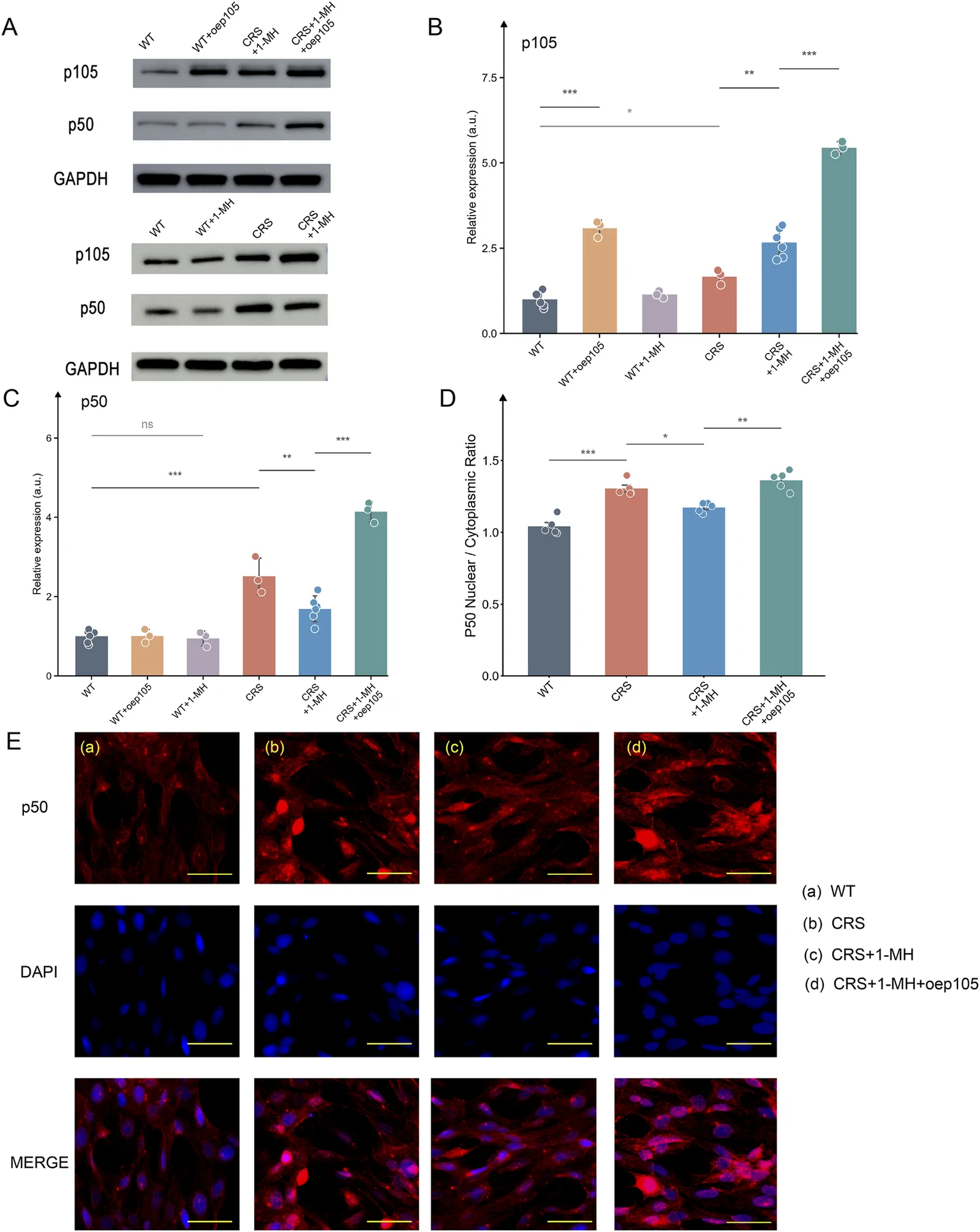
P105 overexpression counteracts the effects of 1-MH on the p105/p50 axis. **(A)** Representative immunoblots showing p105 and p50 protein expression following p105 overexpression in H9c2 cells. Two complementary immunoblot experiments are shown: the upper panel validates p105 overexpression, whereas the lower panel bridges the overexpression model with the primary experimental groups used throughout the study. **(B,C)** Quantification of p105 and p50 abundance, respectively. **(D)** Nuclear-to-cytoplasmic p50 fluorescence ratio in WT, CRS, CRS + 1-MH, and CRS + 1-MH + oe-p105 cells. **(E)** Representative p50 immunofluorescence images with DAPI counterstaining. Shared bridge samples were used for cross-membrane normalization. Immunoblot data represent three independent experiments per group, whereas immunofluorescence data represent five independent experiments per group. Data are shown as mean ± SD and were analyzed by one-way ANOVA with Tukey’s HSD test unless otherwise indicated; *P < 0.05, **P < 0.01, ***P < 0.001, ns, not significant. Oe-p105, p105 overexpression.

### 3.6 p105 overexpression counteracted the anti-inflammatory effects of 1-MH

We overexpressed p105 in H9c2 cells to determine the functional involvement of the p105/p50 axis in the effects of 1-MH. Immunoblotting confirmed increased p105 abundance after p105 overexpression (Figures 7A,B). p105 overexpression raised relative p50 abundance in bridge-normalized immunoblots from 1.69 ± 0.33 in the CRS + 1-MH group to 4.14 ± 0.26 (P < 0.001; Figures 7A,C). p105 overexpression increased the nuclear-to-cytoplasmic p50 fluorescence ratio relative to the CRS + 1-MH group (Figures 7D,E). CRS serum upregulated *Ptgs2*, *Cxcl2*, and *Il1b* mRNA; 1-MH suppressed all three transcripts, and p105 overexpression increased their expression (Figure 8B).

**FIGURE 8:**
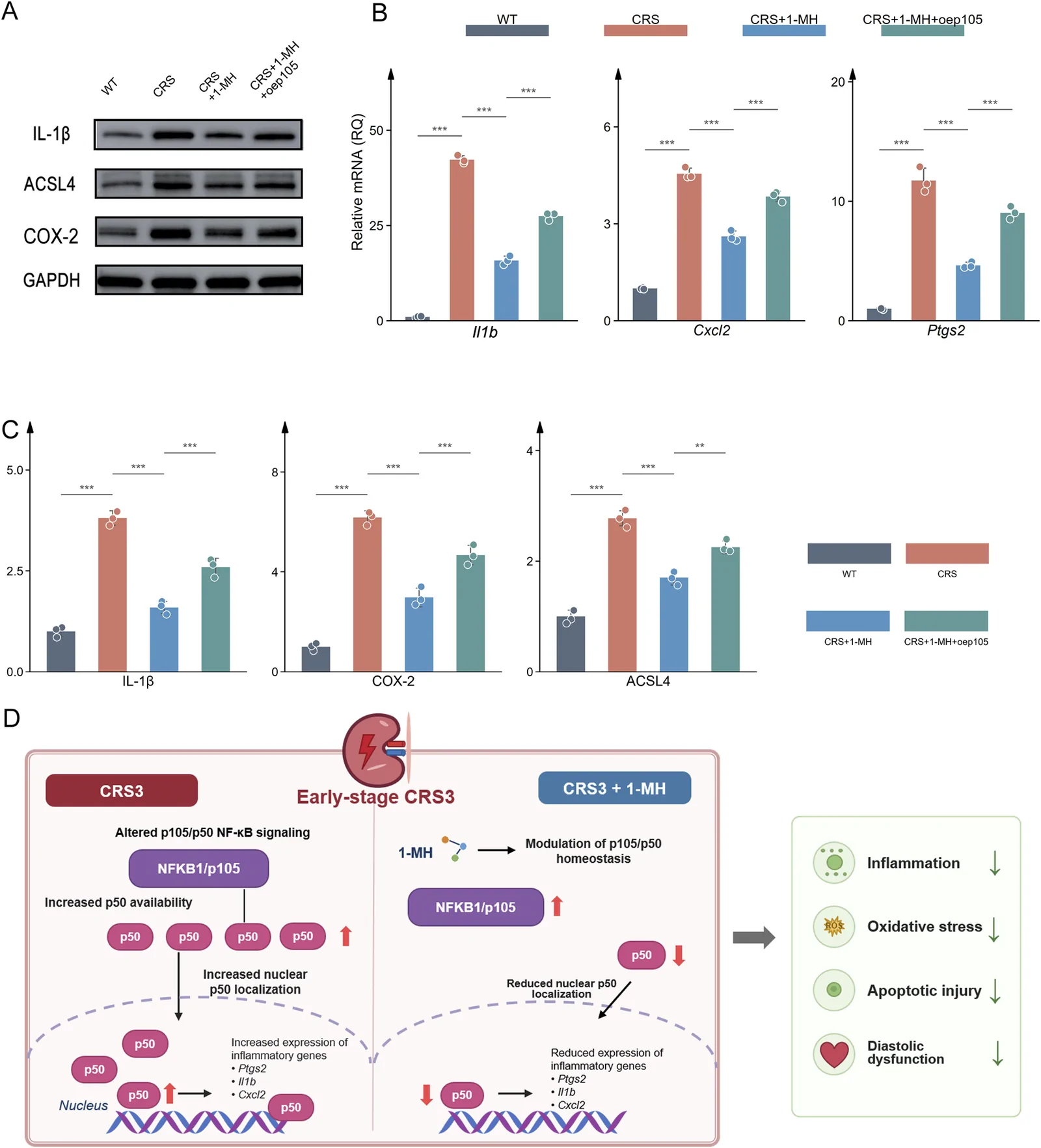
P105 overexpression counteracts the downstream anti-inflammatory effects of 1-MH in CRS serum-exposed H9c2 cells. **(A)** Representative immunoblots showing IL-1β, ACSL4, and COX-2 protein expression. **(B)** Relative mRNA expression of *Il1b*, *Cxcl2*, and *Ptgs2* determined by RT-qPCR. **(C)** Quantification of IL-1β, COX-2, and ACSL4 protein expression. **(D)** Working model illustrating the protective mechanism of 1-MH in early CRS3. Integrated metabolomic profiling identified 1-MH as a metabolite elevated in both serum and myocardium during early CRS3, and subsequent functional analyses demonstrated its protective effects. The model depicts 1-MH modulation of the p105/p50 NF-κB signaling axis together with reductions in inflammatory gene expression, oxidative stress-related changes in CRS serum-exposed H9c2 cells, and early myocardial injury *in vivo*. The gray arrow denotes the overall protective phenotypes associated with 1-MH treatment and does not imply that each downstream phenotype was directly demonstrated to be mediated by the p105/p50 axis. Immunoblot and RT-qPCR data represent three independent experiments per group. Data are shown as mean ± SD and were analyzed by one-way ANOVA with Tukey’s HSD test unless otherwise indicated; *P < 0.05, **P < 0.01, ***P < 0.001, ns, not significant. Full uncropped immunoblot images corresponding to Figures 7, 8 are provided in Supplementary Figure S4.

COX-2, IL-1β, and ACSL4 protein abundance declined after 1-MH treatment and rose after p105 overexpression (Figures 8A,C). COX-2 and IL-1β protein changes tracked their corresponding mRNA changes. p105 overexpression increased p50 abundance and nuclear p50 accumulation and raised the expression of inflammatory transcripts and proteins suppressed by 1-MH. These results demonstrate the functional involvement of the p105/p50 axis in the anti-inflammatory effects of 1-MH (Figure 8D).

## 4 Discussion

Long-term survival among patients with CRS3 requiring intensive care has been reported to be below 50% (Drubel et al., 2022). Effective therapies are limited, and how AKI-induced metabolic disturbances contribute to CRS3 pathogenesis is poorly defined (Sun et al., 2012; Doi and Rabb, 2016; Fox et al., 2019). Here, paired serum and myocardial metabolomics identified 1-MH as a cross-compartment metabolite, and functional experiments demonstrated its protective effects in CRS3. Transcriptomic profiling showed that 1-MH suppressed inflammatory signaling; integration of these findings with target prediction directed our attention to NFKB1/p105. Ensemble structural analysis identified recurrent candidate interaction regions in p105. CETSA findings were consistent with intracellular p105 target engagement by 1-MH. The observed changes in p105 and p50 abundance, as well as the effects of p105 overexpression, further support a functional role for the p105/p50 NF-κB axis in the anti-inflammatory effects of 1-MH.

Fox et al. (2019) detected alterations in myocardial metabolism and oxidative homeostasis as early as 4 h after ischemic AKI, indicating that the cardiac response begins within hours of renal injury. Overt histological injury and structural remodeling, by contrast, have been reported at later stages (Kelly, 2003; Yap and Lee, 2012; Doi and Rabb, 2016; Lee et al., 2018; Faubel, 2023). Sumida et al. (2015) documented myocardial injury and cardiac dysfunction by 72 h. In our study, at 24 h, when BUN and creatinine reached their highest measured levels, myocardial inflammation and diastolic dysfunction were already evident, whereas systolic function was preserved and histological changes remained limited. This time point therefore represents an early phase in which renal injury is well established and cardiac involvement has emerged, but before major structural damage or systolic dysfunction develops.

AKI is accompanied by systemic metabolic remodeling involving both the circulation and distant organs. Metabolomic studies have documented alterations in circulating metabolites in patients with AKI (Sun et al., 2012), and parallel metabolic disturbances have been observed in the heart, lung, and liver (Fox et al., 2019; Ambruso et al., 2021; Baker et al., 2024). More broadly, rather than serving merely as passive markers of tissue injury, metabolites can directly modulate inflammatory signaling. Itaconate, for instance, suppresses inflammation through KEAP1-NRF2 signaling (Mills et al., 2018), exemplifying a protective metabolic response. Conversely, metabolic remodeling can also drive inflammation: Tannahill et al. (2013) showed that succinate stabilizes HIF-1α and increases IL-1β production. Our paired serum and myocardial metabolomic analysis prioritized 1-MH for further investigation, yet its role in CRS3 remained unknown. We therefore examined the effects of exogenous 1-MH. Previous work showed that 1-MH partially restored epithelial and endothelial barrier resistance under methylglyoxal-induced stress (Wetzel et al., 2024), showing biological activity beyond its role as a metabolic marker. In our study, 1-MH attenuated myocardial inflammation in CRS3 rats and oxidative stress-related changes in H9c2 cells exposed to CRS serum. When administered immediately after renal reperfusion, 1-MH also improved diastolic function, with lower myocardial TUNEL positivity and inflammatory cytokine levels.

Myocardial transcriptomic analysis revealed that 1-MH suppressed inflammatory signaling, accompanied by changes in oxidative metabolism. Target prediction integrated with these signatures identified NFKB1/p105 as the top-ranked target, implicating the p105/p50 arm of NF-κB signaling. p105 has dual functions in the canonical NF-κB pathway. It is the precursor of p50 and an IκB-like regulator that retains NF-κB complexes in the cytoplasm (Liou et al., 1992; Rice et al., 1992; Savinova et al., 2009; Cartwright et al., 2016). Processing of p105 yields p50, which can form NF-κB dimers, including p50/RelA, that translocate to the nucleus, bind κB-responsive elements, and regulate inflammatory gene transcription. Shifts in the p105/p50 balance thus alter p50 availability and downstream NF-κB transcriptional activity. 1-MH increased p105 abundance and decreased p50 abundance, shifting the p105/p50 balance toward p105. p105 overexpression increased the NFKB1/p105 pool and total p50 abundance, partially restored nuclear p50 accumulation, and increased inflammatory transcript and protein levels that had been suppressed by 1-MH. This partial restoration of p50 availability and downstream inflammatory signaling supports a functional role for the p105/p50 NF-κB axis in the anti-inflammatory effects of 1-MH.

NFKB1/p105 is a large multidomain protein for which no experimentally resolved full-length structure is available. We therefore examined an ensemble of independently predicted rat, mouse, and human p105 models. Blind docking identified two recurrent candidate interaction regions, C1 and C2. C1 yielded more favorable docking scores across the 60-model ensemble, particularly in rat models, and was prioritized for structural interpretation. In the representative rat model, the RHD-CT and ANK regions associated with C1 showed relatively high local structural confidence, although interdomain PAE indicated remaining uncertainty in their relative orientation. Recurrent C2 contacts involved the ANK, Death, and C-terminal portions of p105, with the C-terminal component showing lower local structural confidence and greater uncertainty in its relative positioning. We therefore retained C2 as a secondary candidate region but interpreted its C-terminal component more cautiously. A representative C1 pose placed 1-MH within the predicted RHD-CT–ANK interdomain region.

This location is functionally notable because the N-terminal Rel homology domain is retained in mature p50, whereas the C-terminal ankyrin-repeat region contributes to both IκB-like regulation and p105 processing (Liou et al., 1992; Rice et al., 1992; Savinova et al., 2009; Cartwright et al., 2016). The location of C1 within this predicted interdomain region therefore provides a plausible structural context for the observed changes in p105 thermal stability and the p105/p50 balance. CETSA also supported intracellular p105 target engagement by 1-MH. The CETSA results, observed changes in p105 and p50 abundance, and effects of p105 overexpression link 1-MH to regulation of the p105/p50 NF-κB axis and subsequent attenuation of inflammatory signaling.

CETSA findings were consistent with intracellular p105 target engagement by 1-MH, but the assay cannot distinguish direct binding from indirect cellular effects. Direct binding to purified p105, binding affinity, and the precise interaction site remain unresolved. As p105-to-p50 processing kinetics were not directly assessed, the abundance changes were interpreted as altered p105/p50 homeostasis rather than evidence of suppressed p105 processing. Structural interpretation is limited by uncertainty in the relative RHD-CT–ANK interdomain orientation and by the static conformations predicted by AlphaFold 3. Although the ensemble reduced dependence on a single model, C1 remains a candidate interaction region rather than a validated binding site. H9c2 cells do not fully recapitulate mature adult cardiomyocytes, and validation in primary cardiomyocytes would strengthen the physiological relevance of the mechanistic findings. The metabolomic and transcriptomic analyses each included four biological replicates per group and were intended primarily for discovery and pathway prioritization. The post-ischemic cohort extended the prophylactic design, but 1-MH was administered immediately after reperfusion and outcomes were assessed only at 24 h. The effects of delayed administration and longer-term cardiac remodeling remain unknown.

## 5 Conclusion

1-MH attenuates early myocardial inflammation in CRS3, reduces oxidative stress-related changes in CRS serum-exposed H9c2 cells, and modulates the p105/p50 NF-κB axis. This work identifies p105 as a molecular link between AKI-associated metabolic remodeling and myocardial inflammatory signaling. More broadly, these findings highlight metabolite-driven regulation of inflammatory signaling as an important component of the early cardiac response to AKI.

## Data Availability

The datasets presented in this study can be found in online repositories. The names of the repository/repositories and accession number(s) can be found below: https://www.ncbi.nlm.nih.gov/geo/, GSE344175.
