# Supplementary material for: 1-Methylhistidine attenuates early myocardial inflammation in type 3 cardiorenal syndrome through modulation of the p105/p50 NF-κB axis
Source: Front Pharmacol. 2026 Oct 14;17:1974807. doi: 10.3389/fphar.2026.1974807 (PMC13714413; doi:10.3389/fphar.2026.1974807)
Supplement: Supplementary file 1 [file DataSheet1.docx]

**Supplementary Data S1**

**Full uncropped Western blot images corresponding to Figures 7 and 8**

Full, uncropped immunoblot images corresponding to the cropped blots presented in Figures 7 and 8 are provided below. Independent experimental replicates are shown separately, with lane identities and molecular-weight information indicated where available. Red rectangles indicate the regions cropped for presentation in the corresponding main-figure panels.

**Figure 7 – Full uncropped Western blot images**

**A. NFKB1/p105 (105 kDa) & NFKB1/p50(50 kDa), corresponding to [Fig. 7A]**

**Replicate 1**

**Lane / group labels:** [1- WT; 2-WT+oep105; 3-CRS+1-MH; 4-CRS+1-MH+oep105]

|  |   -GAPDH  -p105  -p50  30 kDa-  40 kDa-  52 kDa-  66 kDa-  105 kDa-  140 kDa- |
| --- | --- |

**Replicate 2**

|  |   -GAPDH  -p50  -p105  30 kDa-  40 kDa-  52 kDa-  66 kDa-  105 kDa-  140 kDa- |
| --- | --- |

**Replicate 3**

|  |   -GAPDH  -p50  -p105  30 kDa-  40 kDa-  52 kDa-  66 kDa-  105 kDa-  140 kDa- |
| --- | --- |

**B. NFKB1/p105 (105 kDa) & NFKB1/p50(50 kDa), corresponding to [Fig. 7A]**

**Replicate 1**

**Lane / group labels:** [1- WT; 2-WT+1-MH; 3-CRS; 4-CRS+1-MH]

|  |   -GAPDH  -p50  -p105  35 kDa-  35 kDa-  40 kDa-  40 kDa-  55 kDa-  100 kDa-  130 kDa- |
| --- | --- |

**Replicate 2**

**Lane / group labels:** [INSERT GROUP AND LANE INFORMATION]

|  |   -GAPDH  -p50  -p105  35 kDa-  35 kDa-  40 kDa-  40 kDa-  55 kDa-  100 kDa-  130 kDa- |
| --- | --- |

**Replicate 3**

**Lane / group labels:** [INSERT GROUP AND LANE INFORMATION]

|  |   -GAPDH  -p50  -p105  35 kDa-  35 kDa-  40 kDa-  40 kDa-  55 kDa-  100 kDa-  130 kDa- |
| --- | --- |

**Figure 8 – Full uncropped Western blot images**

**A. [COX-2] (~72 kDa), corresponding to [Fig. 8A]**

**Replicate 1**

**Lane labels:** [ 1-WT; 2-CRS; 3-CRS+1-MH; 4-CRS+1-MH+oep105]

| 105 kDa-  52 kDa- |   1 2 3 4  -GAPDH  -COX-2  8 kDa-  13 kDa-  30 kDa-  20 kDa-  52 kDa-  40 kDa-  66 kDa-  195 kDa-  140 kDa- |
| --- | --- |

**Replicate 2**

|  |   1 2 3 4  -GAPDH  -COX-2  20 kDa-  30 kDa-  40 kDa-  52 kDa-  66 kDa-  105 kDa-  195 kDa-  140 kDa- |
| --- | --- |

**Replicate 3**

|  | 1 2 3 4  -COX-2  52 kDa-  66 kDa-  105 kDa-  140 kDa-  195 kDa- |
| --- | --- |

-GAPDH

20 kDa-

30 kDa-

40 kDa-

**B. [ACSL4] (79kDa), corresponding to [Fig. 8A]**

**Replicate 1**

**Lane / group labels:** [1-WT; 2-CRS; 3-CRS+1-MH; 4-CRS+1-MH+oep105]

|  |   1 2 3 4  -ACSL4  -GAPDH  30 kDa-  40 kDa-  52 kDa-  66 kDa-  105 kDa- |
| --- | --- |

**Replicate 2**

|  |   1 2 3 4  -ACSL4  -GAPDH  30 kDa-  52 kDa-  40 kDa-  66 kDa-  105 kDa- |
| --- | --- |

**Replicate 3**

|  |   1 2 3 4  -ACSL4  -GAPDH  30 kDa-  105 kDa-  66 kDa-  52 kDa-  40 kDa- |
| --- | --- |

**C. [IL-1β] (35kDa), corresponding to [Fig. 8A]**

**Replicate 1**

**Lane / group labels:** [1-WT; 2-CRS; 3-CRS+1-MH; 4-CRS+1-MH+oep105]

|  |   -IL-1β  -GAPDH  30 kDa-  40 kDa-  52 kDa-  105 kDa- |
| --- | --- |

**Replicate 2**

|  |   -IL-1β  -GAPDH  30 kDa-  40 kDa-  52 kDa-  105 kDa- |
| --- | --- |

**Replicate 3**

|  |   -IL-1β  -GAPDH  30 kDa-  40 kDa-  52 kDa-  105 kDa- |
| --- | --- |

**Figure 6 – Full uncropped Western blot images**

**A. NFKB1/p105 (105 kDa), corresponding to [Fig. 6F]**

**Replicate 1-3(50μm)**

**Lane / group labels:** [1- RT; 2-40℃; 3-45℃; 4-50℃; 5-55℃; 6-60℃]

p105-

p105-

130 kDa-

-130 kDa

-130 kDa

-100 kDa

-p105

-100 kDa

100 kDa-

**Replicate 1-3(100μm)**

100 kDa-

100 kDa-

100 kDa-

130 kDa-

130 kDa-

130 kDa-

-p105

-p105

-p105

**Replicate 1-3(Vehicle)**

-p105

-p105

p105-

-100 kDa

130 kDa-

130 kDa-

-130 kDa

100 kDa-

100 kDa-
