# Supplementary material for: 1-Methylhistidine attenuates early myocardial inflammation in type 3 cardiorenal syndrome through modulation of the p105/p50 NF-κB axis
Source: Front Pharmacol. 2026 Oct 14;17:1974807. doi: 10.3389/fphar.2026.1974807 (PMC13714413; doi:10.3389/fphar.2026.1974807)
Supplement: Supplementary file 2 [file Supplementaryfile1.docx]

# **Supplementary Figure S1.**

**Relative abundance of 1-methylhistidine in myocardium and serum from Sham and CRS rats.**

Relative 1-methylhistidine abundance was increased in both myocardium and serum in the CRS group compared with the Sham group. Data are presented as mean ± SD. Each point represents an individual sample. *P < 0.05 and **P < 0.01 versus Sham.

# **Supplementary Figure S2.**

**Ensemble docking stability, residue-contact mapping, and AlphaFold 3 structural-confidence analyses of candidate interaction regions in NFKB1/p105.**

(A) Technical stability of focused docking for C1 and C2 across the 60-model ensemble. For each receptor–region pair, 10 independent AutoDock Vina runs were performed using different random seeds. The main plot shows the range of the 10 top-ranked docking scores, and the inset shows the corresponding interquartile range (IQR). (B) Contact frequencies of selected C1-associated residues across the full 60-model ensemble. Ligand–protein contacts were defined by a minimum heavy-atom distance of ≤4.0 Å using the top-ranked pose from a fixed-seed (seed = 0) focused-docking run for each model. Homologous mouse and human residues were mapped to rat reference numbering. (C) Species-stratified contact frequencies for the selected C1-associated residues in rat (n = 30), mouse (n = 15), and human (n = 15) models. (D) Representative AlphaFold 3 predicted aligned error (PAE) map of a rat NFKB1/p105 model. (E) Model-wise bidirectional mean PAE between the RHD-CT (residues 241–400) and ANK (residues 545–743) regions across the 60-model ensemble. For each model, the mean PAE values for the RHD-CT→ANK and ANK→RHD-CT residue pairs were averaged to obtain the bidirectional interdomain PAE. Each colored point represents one model. Horizontal bars indicate the median, and whiskers indicate the interquartile range (Q1–Q3). (F) Per-residue predicted local distance difference test (pLDDT) profile of the same representative rat NFKB1/p105 model shown in panel D. The RHD-CT and ANK regions are indicated, together with the selected C1-associated residues Asp303, Ser682, Arg684, and Thr718. PAE reflects confidence in relative residue positioning, whereas pLDDT reflects local structural confidence. ANK, ankyrin-repeat region; PAE, predicted aligned error; pLDDT, predicted local distance difference test; RHD-CT, C-terminal portion of the Rel homology domain.

# **Supplementary Figure S3.**

**Protective effects of post-ischemic 1-MH administration in early CRS3.**

A separate post-ischemic cohort was established in which rats were assigned to Sham, CRS, and CRS + 1-MH groups (n = 5 per group). 1-MH (20 mg/kg, intravenous) was administered immediately after renal reperfusion, and outcomes were assessed at 24 h. (A) Quantification of the E/A ratio, fractional shortening (FS), and left ventricular ejection fraction (LVEF). (B) Representative myocardial TUNEL fluorescence images showing DAPI, TUNEL, and merged channels. Scale bars, 100 μm. (C) Quantification of TUNEL-positive myocardial cells. (D, E) Myocardial IL-6 and TNF-α levels measured by ELISA and normalized to total protein, respectively. All five animals per group were included in the analyses. ELISA measurements were performed in technical triplicate, and the triplicate mean was treated as one biological data point per animal. Data are presented as mean ± SD and were analyzed by one-way ANOVA followed by Tukey’s HSD test; *P < 0.05, **P < 0.01, ***P < 0.001; ns, not significant. This post-ischemic cohort was analyzed independently from the prophylactic cohort, and no statistical comparisons were performed between cohorts.

# **Supplementary Table S1.**

**Primer sequences used for RT-qPCR analysis.**
Primer sequences used for quantitative reverse-transcription PCR (RT-qPCR) analysis of *Ptgs2, Cxcl2,* and *Il1b* are listed, including the corresponding forward and reverse primer sequences.

# **Supplementary Table S2.**

Plasmid information used for p105 overexpression experiments.
Details of the rat *Nfkb1*/p105 overexpression plasmid and the corresponding control vector used in H9c2 cells are provided, including the relevant construct and vector information.

# **Supplementary Table S3.**

**SuperPred target-prediction results for 1-MH.**

For each predicted target, the table lists the ChEMBL identifier, UniProt identifier, available PDB entries, Therapeutic Target Database (TTD) identifier, prediction probability, and corresponding model accuracy.

# **Supplementary Table S4.**

**Per-model focused-docking stability, residue contacts, and RHD-CT–ANK interdomain PAE for the NFKB1/p105 structural ensemble.**

For each of the 60 independently generated AlphaFold 3 NFKB1/p105 structural models, the table reports the median, minimum, maximum, range, and interquartile range (IQR) of 10 repeated focused-docking scores for C1 and C2; the C1 minus C2 score difference (Δscore); ligand-contacting residues mapped to rat reference numbering; the bidirectional mean predicted aligned error (PAE) between the RHD-CT and ANK regions associated with C1; and bidirectional mean PAE values for the ANK–Death, ANK–C-terminal, and Death–C-terminal region pairs associated with C2. Summary local pLDDT values for recurrent C2 contact residues and selected C1-associated residues are also provided. The 10 docking runs for each receptor–region pair were treated as technical replicates. For contact analysis, the top-ranked pose from a fixed-seed (seed = 0) focused-docking run was used for each receptor–region pair. Ligand–protein contacts were defined using a minimum heavy-atom distance cutoff of ≤4.0 Å. Negative Δscore values indicate more favorable docking scores for C1 than for C2. Docking scores are reported in kcal/mol and PAE values in Å. ANK, ankyrin-repeat region; C1/C2, candidate interaction regions 1 and 2; IQR, interquartile range; PAE, predicted aligned error; pLDDT, predicted local distance difference test; RHD-CT, C-terminal portion of the Rel homology domain.
